# Extraction, Enrichment, Characterization, and Antioxidant Activities of *Sargassum fusiforme* Polyphenols

**DOI:** 10.3390/foods14193317

**Published:** 2025-09-24

**Authors:** Hui Wang, Min Zhang, Weiqin Yang, Linwu Zhuang, Lei Guo

**Affiliations:** 1 Jiangsu Key Laboratory of Marine Bioresources and Environment, School of Ocean Food and Biological Engineering, Jiangsu Ocean University, Lianyungang 222005, China; 2022220859@jou.edu.cn (H.W.); 2023122089@jou.edu.cn (M.Z.); 2 Lianyungang Customs Comprehensive Technology Center, Lianyungang 222042, China; 3 Lianyungang Quality and Technology Comprehensive Inspection and Testing Center, Lianyungang 222000, China; 2019220128@jou.edu.cn

**Keywords:** *Sargassum fusiforme*, polyphenols, extraction, characterization, antioxidant activity

## Abstract

This study investigated the extraction, enrichment, characterization, and antioxidant activities of *Sargassum fusiforme* polyphenols (SFPs). The optimal extraction process conditions of SFPs are as follows: an ethanol volume fraction of 28%, a liquid–solid ratio of 29 mL/g, an extraction temperature of 80 °C, and an extraction time of 3.2 h. After enrichment by D101 macroporous resin, the purity of SFPs increased from 1.20 ± 0.08% to 10.78 ± 0.25%, increasing by approximately 8 times. SFPs were mainly composed of polyphenols, flavonoids, and polysaccharides. Furthermore, after identification by HPLC-QQK-ESI-MS/MS, they were found to contain 11 phlorotannins (mainly of the fuhalol type), 2 flavonoids, etc. In three antioxidant evaluation systems (DPPH free radical scavenging ability, reducing power, and total antioxidant capacity), the enriched SFPs all exhibited superior activities compared to tea polyphenols. The research results provide a theoretical basis and experimental evidence for the development of a new type of food preservative based on SFPs.

## 1. Introduction

*Sargassum fusiforme* (Harvey) Setchell, synonym *Hizikia fusiformis* (Harvey) Okamura, also known as Yangqicai in Chinese, Tot in Korean, and Hiziki in Japanese, is a kind of brown algae that can be used both as medicine and food. It belongs to the class Phaeophyceae, order Fucales, and family Sargassaceae [1]. *Sargassum fusiforme* grows in large quantities along the rocky coastlines of Asian countries such as China, South Korea, and Japan. The earliest record of the use of *Sargassum fusiforme* as a traditional Chinese medicine is in “*Shennong’s Classic of Materia Medica*” in 200 AD. It is called seaweed and is famous for treating tumor-like hard lumps, difficulty in urination, and edema [2,3]. At present, *Sargassum fusiforme* has been successfully farmed in southern China and Korea. In 2020, China raised 26,533 tons annually, making it one of the world’s largest producers of *Sargassum fusiforme* [4].

*Sargassum fusiforme* is rich in polysaccharides, polyphenols, dietary fiber, protein, trace elements, etc., and is widely used in traditional cooking [5]. Meanwhile, due to its economic value in the pharmaceutical and manufacturing industries, research on the active ingredients and medical effects of *Sargassum fusiforme* has been increasing in recent years. The bioactive components of *Sargassum fusiforme* that can be explored for practical applications mainly include polysaccharides (such as alginate and fucoidan) and polyphenols [6]. The polyphenols of *Sargassum fusiforme* (SFPs) mainly include phlorotannins, phenolic acids, and flavonoids [7,8]. In particular, phlorotannins are a type of polyphenolic substance that exists only in brown algae [9]. They are polymeric from phloroglucinol (1,3,5-trihydroxybenzene) and participate in many important secondary metabolic processes, such as chemical defense and prevention of oxidative damage caused by changes in nutritional availability and ultraviolet radiation [10].

Brown algae polyphenols are non-toxic secondary phytochemicals with extensive biological potentials such as antioxidant, antibacterial, and anti-inflammatory properties [9,11]. The antioxidant activity especially makes brown algae polyphenols have broad application prospects in the fields of food, cosmetics, and medicine [12,13,14]. As an edible seaweed, although *Sargassum fusiforme* is rich in polyphenols, the exploration of its practical application in food preservation is still far from sufficient. Therefore, the aim of this study is to optimize the extraction and enrichment process of SFPs, identify its phenolic compounds, and evaluate its antioxidant activities, providing a theoretical basis and experimental evidence for the development of new food preservatives based on SFPs.

## 2. Materials and Methods

### 2.1. Materials

*Brown seaweed Sargassum fusiforme* was purchased from an aquatic product store in Xiapu, Ningde City, Fujian Province. After being crushed by a multi-functional grinder and passed through a 40-mesh sieve, the fine powder obtained was sealed and stored in a dry and cool environment for future use. 1,1-Diphenyl-2-picrylhydrazyl radical (DPPH), gallic acid, rutin, and L-fucose were purchased from Hefei Bomei Biotechnology Co., LTD (Hefei, China). Tea polyphenols were purchased from Shanghai Yuanye Biotechnology Co., LTD (Shanghai, China). The total antioxidant capacity (T-AOC) determination kit was purchased from Nanjing Jiancheng Bioengineering Research Institute (Nanjing, China). Macroporous adsorption resins (AB-8, HP20, CAD40, D101) were purchased from Shanghai Xinhu Experimental Equipment Co., LTD (Shanghai, China). Biochemical reagents such as ethanol, phenol, sulfuric acid, and Folin and Ciocalteu’s phenol reagent were purchased from Sinopharm Chemical Reagent Co., LTD (Shanghai, China).

### 2.2. Single-Factor Experiments

In total, 1.0 g of *Sargassum fusiforme* powder was weighed and mixed with a certain volume of ethanol aqueous solution, then added to a 250 mL flat-bottomed flask. The flask was then placed in a water bath for heating and soaking for extraction. The specific parameters are shown in Table 1. After the extraction was completed, the filtrate was obtained by suction filtration using a Buchner funnel, and the ethanol was removed by vacuum concentration at 55 °C. Then, it was transferred to a volumetric flask with pure water and made up to 25 mL. The total phenol content was determined. The crude extract was diluted with pure water to the specified concentration based on the calculated TPC, and the radical scavenging rate of DPPH (10 mg/L) and the radical scavenging rate of hydroxyl (50 mg/L) were determined.

### 2.3. Box–Behnken Design

The Box–Behnken design and response surface methodology were used to optimize the extraction process of SFPs. Based on the results of the single-factor experiments, with the volume fraction of ethanol (*X*_1_), liquid–solid ratio (*X*_2_), and extraction time (*X*_3_) as independent variables, taking the content of polyphenols (*Y*_1_) and the clearance rates of hydroxyl radicals (*Y*_2_) and DPPH radicals (*Y*_3_) as response variables, a Box–Behnken design was adopted to construct a scheme with three factors (*X*_1_, *X*_2_, *X*_3_) and three levels (−1, 0, +1), totaling 17 experiments conducted. Subsequently, the Design Expert 8.0.6 software was used to analyze data, establish models, and predict the optimal combination.

### 2.4. Determination of Crude SFP Extract

#### 2.4.1. Total Phenol Content

The content of polyphenols was determined by Folin and Ciocalteu’s phenol method [15]. In total, 1.0 mL of the SFP solution was taken into a 10 mL volumetric flask; then, 1.25 mL Folin and Ciocalteu’s phenol reagent and 4 mL of 7.5 g/100 mL sodium carbonate were added and made up to 10 mL with distilled water. The reaction system was transferred into a 30 °C water bath and covered. After 60 min of incubation, the completed chromogenic complex was dark blue. The absorbance at 760 nm was detected using a UV spectrophotometer. The standard curve was made with gallic acid as the standard substance, and the content of polyphenols was expressed as the gallic acid equivalent (mg GAE/g DW).

#### 2.4.2. DPPH Radical Scavenging Activity

The determination of the DPPH radical scavenging activity referred to the method in [16] and made appropriate adjustments. In short, 2 mL of 0.2 mM DPPH radical anhydrous ethanol solution was mixed with 2 mL of the sample at room temperature in the dark. After an accurate reaction for 30 min, the absorbance at 517 nm was measured (*A_i_*), and pure water was used as the blank reference (*A_c_*). Due to the dark background color of the extract, it is necessary to measure the absorbance of 2 mL of the corresponding concentration of the analyte and 2 mL of anhydrous ethanol at 517 nm (*A_j_*) to remove background interference. The calculation formula for the radical scavenging rate (*Y*) of DPPH is as follows:

(1) $$Y\;=\;\left(1\;-\;\frac{A_{i}-A_{j}}{A_{c}}\right)\times 100\%$$

#### 2.4.3. Hydroxyl Radical Scavenging Activity

The determination of the hydroxyl radical scavenging activity referred to the method in [17] and made appropriate adjustments. In a test tube, 1 mL each of the aqueous solution of the substance to be tested, FeSO_4_ solution (9 mM), salicylic acid–ethanol solution (10 mM), and hydrogen peroxide solution (8.8 mM) was added in sequence. The solutions were quickly mixed and incubated in a 37 °C water bath for 20 min. The absorbance (*A_i_*) at 508 nm was detected. According to the above operation, distilled water was used instead of the sample solution to determine *A*c, and distilled water was used instead of the H_2_O_2_ solution to determine *A_j_*. The hydroxyl radical scavenging rate (*Y*) was calculated according to the above Equation (1).

### 2.5. Enrichment of SFPs by Macroporous Resin

#### 2.5.1. Determination of Static Adsorption and Desorption Capacity

In total, 1.0 g (*W*) of each of the four pre-treated macroporous resins (AB-8, HP20, CAD40, D101) was weighed and placed in centrifuge tubes. Then, 20 mL (*V_i_*) of crude SFP extract was added, and the initial total phenol concentration (*C*_0_) was determined as described in Section 2.4.1. In this experiment, a crude SFP extract solution with a concentration of 1.89 mg/mL was used. The centrifuge tubes were placed on a shaker and shaken at room temperature in the dark for 12 h, and the total phenol concentration (*C*_1_) of the supernatant was determined. The adsorption capacity (mg GAE/g) was calculated according to the following formula:

(2) $$Q_{e}\;=\;\frac{(C_{0}-C_{1})V_{i}}{W}$$

The four macroporous resins that were adsorbed were washed three times with pure water and placed in centrifuge tubes, and 20 mL of ethanol–water solution (50%) was added to elute the adsorbed phenolic substances. They were then placed on a shaker and shaken at room temperature in the dark for 12 h to complete desorption. The volume (*V_d_*) and total phenol concentration (*C_d_*) of the filtrate were determined, and the desorption capacity (mg GAE/g) and desorption rate (%) were calculated according to the following formulas:

(3) $$Q_{d}\;=\;\frac{C_{d}V_{d}}{W}$$

(4) $$D\;\left(\%\right)=C_{d}\times \frac{C_{d}V_{d}}{\left(C_{0}-C_{1}\right)V_{i}}$$

#### 2.5.2. Drawing of the Adsorption Kinetics Curve

In total, 1.0 g of macroporous resin (AB-8, HP20, D101) was weighed and added to 20 mL of crude SFP extract (2.06 mg GAE/mL); the centrifuge tubes were placed on a shaker and shaken at room temperature in the dark. Samples were taken at different time points (1, 2, 3, 4, 6, 8, 10, 12 h) after oscillation to determine the total phenol concentration of the supernatant. The adsorption amounts at different time points were calculated. A line graph was plotted with time as the abscissa and adsorption amount as the ordinate.

#### 2.5.3. Screening of Sample Loading Concentration, Flow Rate, and Volume

In total, 1.0 g of the dried D101 macroporous resin was accurately weighed and added to different concentrations (0.5, 1, 2, 3, and 4 mg GAE/mL) of crude SFP extracts at 40 mg each, that is, 80, 40, 20, 13.3, and 10 mL, respectively. The mixtures were shaken in the dark at room temperature for 6 h on a shaker. The polyphenol adsorption amounts at different concentrations of crude SFP extracts were calculated to analyze the influence of different concentrations of crude SFP extracts on the adsorption of macroporous resin and to obtain the optimal sample loading concentration. The treated D101 macroporous resin was packed into a glass chromatography column (2.5 × 30 cm) by the wet packing method, with a height of half of the column length. The crude SFP extract with a concentration of 2 mg GAE/mL was loaded at flow rates of 1, 2, and 4 BV/h. The total phenol content of the effluent was detected at a frequency of 0.5 BV/time. After deducting the dead volume, the leakage curve was drawn based on the polyphenol concentration of the effluent, and the optimal loading flow rate and loading volume were determined.

#### 2.5.4. Screening of Volume Fraction, Flow Rate, and Volume of Ethanol Elution Solution

In total, 1.0 g of saturated adsorption D101 macroporous resin was weighed and placed in a centrifuge tube. In total, 20 mL of ethanol solutions of different volume fractions (30%, 40%, 50%, 60%, and 70%) was added. Static desorption was carried out for 1 h using a shaker. The desorption solution was concentrated by rotary evaporation to remove ethanol, and the total phenol content was determined. This analysis was used to determine the optimal concentration of the desorption solvent. The chromatographic column after adsorption was rinsed with pure water until the effluent was colorless. The liquid level was discharged to the column bed height, and 40% ethanol solution was slowly added for elution at flow rates of 2, 3, and 4 BV/h. The total phenol content of the effluent was detected at a frequency of 0.5 BV/time. After removing the dead volume, the desorption curve was plotted with the effluent volume as the abscissa and the polyphenol concentration as the ordinate to determine the flow rate and optimal dosage of the eluent.

### 2.6. Characterization assays of SFPs

#### 2.6.1. Total Flavonoid Content

The determination of the total flavonoid content was carried out by the colorimetric method [18], using rutin as the standard to prepare a gradient working solution (0, 20, 40, 60, 80, 100 μg/mL). In total, 2 mL of the test solution or standard working solution was taken and placed in a test tube. Then, 0.3 mL of sodium nitrite solution (5%), 0.3 mL of aluminum nitrate solution (10%), and 4 mL of sodium hydroxide solution (4%) were added successively. After a full reaction for 15 min, the carbonyl and hydroxyl groups formed an orange-yellow complex with aluminum in an alkaline environment. The absorbance at 510 nm was measured, and a standard curve was plotted with rutin concentration as the abscissa and absorbance as the ordinate. The total flavonoid content was calculated based on the standard curve.

#### 2.6.2. Polysaccharide Content

The determination of the total sugar content was carried out by the phenol-concentrated sulfuric acid method [19], and glucose was used as the standard to prepare gradient working solutions (0, 20, 40, 60, 80, 100 μg/mL). In total, 2 mL of the test solution or standard working solution was transferred and placed in a test tube. After adding 1 mL of phenol solution (5%), 5 mL of concentrated sulfuric acid was slowly added. The reaction was fully carried out at room temperature for 30 min. Under the action of concentrated sulfuric acid, the polysaccharide undergoes hydrolysis, and the monosaccharide produced reacts with phenol to form an orange-yellow compound. After cooling to room temperature, the absorbance at 490 nm was detected, and a standard curve was plotted with the glucose concentration as the abscissa and the absorbance as the ordinate. In this experiment, the interference of the original base color of the SFP extract also needs to be deducted. The content of total sugar was calculated through the standard curve.

#### 2.6.3. Fucose Content

The determination of fucose content was conducted by the colorimetric method optimized by Gibbons [20] based on the Dische method. L-fucose was used as the standard to prepare gradient working solutions (0, 20, 30, 40, 50, 60, 70 μg/mL). In total, 4.5 mL of sulfuric acid solution (87%) was added to 1 mL of the test solution or standard working solution. After cooling to room temperature in ice water and incubating at 100 °C for 10 min, 0.1 mL of L-cysteine hydrochloride solution (3%) was added and incubated at 37 °C again for 90 min. The absorbance at 396 nm and 427 nm was measured. A fitting equation was established with the L-fucose concentration as the abscissa and the difference in absorbance at 396 nm and 427 nm as the ordinate, and the fucose content of the analytes was calculated from it. L-fucose is a deoxyhexose; the yellow product formed by it and L-cysteine hydrochloride has a strong absorbance at 396 nm but no absorbance at 427 nm. The absorbance of the reaction product of the interfering component hexose at these two wavelengths is similar. This detection method can remove the interference and accurately quantitatively detect fucose in the substance to be tested.

#### 2.6.4. HPLC-QQQ-ESI-MS/MS Qualitative Analysis

The enriched SFPs were dissolved in ultrapure water, passed through a 0.22 μm filter membrane, and then detected by AB Sciex QTRAP^TM^ 4500 LC/MS/MS (Danaher Corporation, Shanghai, China). The parameters for HPLC were as follows: Sample separation was carried out using ACE’s C18 chromatographic column (2.6 μm, 2.1 × 100 mm), and the column oven was set to 40 °C. The aqueous phase (A) was 0.1% formic acid aqueous solution, and the organic phase (B) was methanol. The flow rate was 0.3 mL/min. An isocratic elution of 20/80 (A: B, *v*/*v*) was used, and the monitoring pressure was 3–800 bar. The temperature of the injection chamber was 4 °C, and the injection volume was 20 μL.

The parameters for the mass spectrometry analysis experiment were set as follows: An ESI ion source was used in the negative ion mode under the multiple reaction monitoring (MRM) mode for specified mass monitoring. The mass-to-charge ratio information of the parent ions and daughter ions was derived from the relevant literature [8,10,20]. The declustering potential (DP) was 60–80 V, and the collision energy (CE) was 20–40 V, which need to be optimized according to the actual usage conditions. The dwell time was 20–50 ms, and it should be adjusted based on at least 12 monitoring points for each peak (calculated based on the cycle) and a total scan time (total scan time) of no more than 0.8 s. The curtain gas pressure (CUR) was 30 psi, the collision gas pressure (CAD) was 8 psi, and the ion source voltage (IS) was 5000 V.

### 2.7. Antioxidant Activities of SFPs and Tea Polyphenols

#### 2.7.1. Radical Scavenging Activities

The operation steps for determining the scavenging activities of the DPPH radical and hydroxyl radical are the same as those in Section 2.4.2 and Section 2.4.3.

#### 2.7.2. Reducing Ability

The determination of reducing ability was carried out according to the Prussian blue method [21]. In total, 1 mL of the aqueous solution of the sample was successively added with 1 mL of PBS (0.2 mol/L, pH = 6.6) and 1 mL of potassium ferricyanide (1%). After a full reaction at 50 °C for 20 min, 1 mL of the supernatant was taken and mixed with 1 mL of pure water and 0.5 mL of ferric chloride (0.1%). After a reaction for 10 min, the absorbance at 700 nm was detected. The greater the absorbance, the stronger the reducing ability.

#### 2.7.3. Total Antioxidant Capacity

The determination of the total antioxidant capacity of SFPs was carried out using the Total Antioxidant Capacity Assay Kit (Phenanthroline Method, Nanjing Institute of Bioengineering, Nanjing, China) reported in the literature [22]. Briefly, 1 mL, 2 mL, and 0.5 mL of reagents 1, 2, and 3 were, respectively, added to the testing tube and control tube. Then, 0.1 mL of the testing sample was added only to the testing tube. After thorough mixing in the vortex mixer, the tubes were bathed in a 37 °C water bath for 30 min. In total, 0.1 mL of reagent 4 was added to the testing tube and control tube; then, 0.1 mL of the testing sample was added only to the control tube. The testing tube and control tube were further mixed and bathed for 10 min, the absorbance was measured at 520 nm by the TU-1901 UV-Vis Spectrophotometer (Beijing Persee General Instrument Co., LTD., Beijing, China), and the total antioxidant capacity was calculated and expressed in U/mL.

### 2.8. Statistical Analysis

All the experiments were conducted in parallel three times, and the data are expressed as the mean ± standard deviation. Statistical comparisons were performed with the one-way ANOVA statistical test, and *p* values < 0.05 were considered statistically significant.

## 3. Results and Discussion

### 3.1. Optimization of Extraction Process of SFPs with Antioxidant Activity

**Figure 1:**
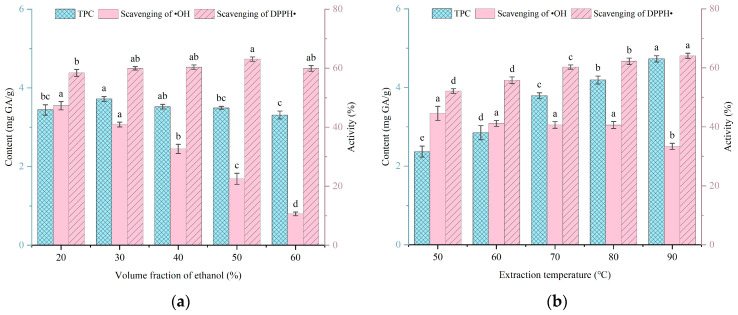

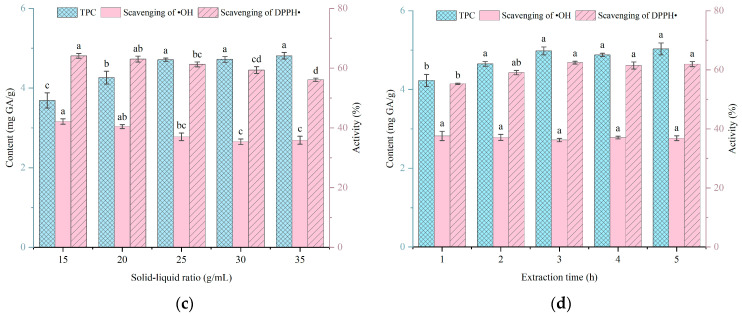
Effects of (**a**) ethanol volume fraction, (**b**) extraction temperature, (**c**) liquid–solid ratio, and (**d**) extraction time on the content of polyphenols, hydroxyl radical scavenging activity, and DPPH radical scavenging activity of crude SFP extract. The letters a–d indicated a significant difference (*p* < 0.05).

In the single-factor experiment of extracting SFPs with antioxidant activity in an ethanol aqueous solution, the effects of the ethanol volume fraction, extraction temperature, liquid–solid ratio, and extraction time on the total phenol content and hydroxyl radical and DPPH radical scavenging activities in the crude SFP extract were investigated. As shown in Figure 1a, 30% ethanol can form relatively stable hydrogen bonds with specific polar phenolic hydroxyl groups and extract polyphenols from cells. Due to the polarity of phenolic substances being influenced by the number, position, and structure of phenolic hydroxyl groups [23], the optimal ethanol volume fraction used by researchers in different reports may vary. However, generally speaking, polyphenols are suitable for medium polarity. The crude polyphenol extract obtained by ethanol aqueous solution extraction may contain various active substances such as flavonoids, polysaccharides, terpenes, and unsaturated fatty acids [8]. Moreover, due to the low specificity of Folin and Ciocalteu’s method for detecting polyphenols, some reducing substances may be identified as polyphenols [24]. Under the combined effect of these substances, the relationship between the two dependent variables, the total phenol content and DPPH scavenging rate, and the ethanol volume fraction is not significant. In contrast, under the influence of the combined effect and low specificity, a certain type of specific substance that scavenges hydroxyl radicals needs to be extracted through stronger polarity, which is manifested as a significant decrease in the hydroxyl radical scavenging rate with the increase in the ethanol volume fraction. Considering the comprehensive extraction rate, 20%, 30%, and 40% ethanol volume fractions were selected for optimization experiments.

As shown in Figure 1b, as the temperature increases, that is, the effective diffusion rate increases, the extraction rate of cellular contents significantly increases. The total phenolic content and DPPH radical scavenging rate are significantly affected by temperature, but a temperature of 90 °C causes oxidation of active substances, resulting in a decrease in the hydroxyl radical scavenging rate. Since the boiling point of pure ethanol is 78.3 °C, the sealed space for extraction follows Henry’s law and Raoult’s law. The extraction conditions are not ideal, and the boiling point of the ethanol–water mixture will not be the same as the theoretical value, but it will still be higher than that of pure substances. The boiling point of 20% to 40% ethanol solutions is approximately 83.1–87.0 °C. To save costs and reduce operational difficulty, the condensation reflux required for extraction at 90 °C is omitted, and the next experiment is conducted at a fixed temperature of 80 °C.

As shown in Figure 1c, the total phenol content reaches its peak when the liquid–solid ratio is 25 mL/g. Subsequently, further increasing the solvent volume does not increase the extraction amount. Instead, due to the competition for dissolution by impurities, the solvent may absorb impurities such as proteins and lipophilic compounds [16], causing the antioxidant capacity to continuously decrease with the increase in the solvent. Excessive solvents will also increase the time cost of vacuum concentration. Therefore, 20 mL/g, 25 mL/g, and 30 mL/g were selected for the optimization experiment.

As shown in Figure 1d, the total phenolic content and antioxidant activity were significantly related to time within 3 h. After that, the system remained in a stable state, with no more substances flowing out and no changes in activity due to the extension of time, tending to be stable overall. Therefore, an extraction time of 3 h is sufficient. The hydroxyl radical scavenging rate was not significantly affected by time, indicating that the significant influencing factors for it are temperature and the ethanol volume fraction. Therefore, optimization experiments were conducted at 2 h, 3 h, and 4 h.

Furthermore, the temperature of the optimization step was fixed at 80 °C, and the three independent variables were the volume fraction of ethanol (*X*_1_), the liquid–solid ratio (*X*_2_), and the extraction time (*X*_3_). The three dependent variables were the total phenol content (*Y*_1_), the hydroxyl radical scavenging rate of 50 mg/L crude SFP extract (*Y*_2_), and the DPPH radical scavenging rate of 10 mg/L crude SFP extract (*Y*_3_). The matrix and experimental results of the Box–Behnken experiment design are shown in Table 2.

The results of the analysis of variance, as shown in Table 3, reflect whether the established optimization model is reliable. The *p* values of *Y*_1_, *Y*_2_, and *Y*_3_ are all less than 0.01, indicating that the established models are significant. Meanwhile, their lack of fit is not significant (*p* > 0.05). The *R*^2^ values are 0.9610, 0.9344, and 0.9444, respectively, all close to 1, indicating a good fit and the ability to predict the actual situation very well. The coefficient of variation (C.V.%) values are all less than 5, indicating that the credibility and accuracy of the experiment are both very high.

The regression equations obtained through modeling with the Design Expert software (version 8.0.6) are as follows:

*Y*_1_ = 5.05 + 0.094 *X*_1_ + 0.20 *X*_2_ + 0.12 *X*_3_ − 0.098 *X*_1_*X*_2_ − 0.071 *X*_1_*X*_3_ − 0.12 *X*_2_*X*_3_ − 0.22 *X*_1_^2^ + 0.15 *X*_2_^2^ − 0.15 *X*_3_^2^;

*Y*_2_ = 36.74 − 3.19 *X*_1_ + 0.011 *X*_2_ + 0.098 *X*_3_ + 0.51 *X*_1_*X*_2_ − 0.095 *X*_1_*X*_3_ + 0.16 *X*_2_*X*_3_ − 2.20 *X*_1_^2^ + 0.47 *X*_2_^2^ − 2.62 *X*_3_^2^;

*Y*_3_ = 62.87 + 0.42 *X*_1_ + 0.55 *X*_2_ + 1.21 *X*_3_ + 0.55 *X*_1_*X*_2_ − 1.04 *X*_1_*X*_3_ + 0.45 *X*_2_*X*_3_ − 1.61 *X*_1_^2^ – 0.38 *X*_2_^2^ − 1.67 *X*_3_^2^.

Table 3 lists the influence of the three independent variables on the results. All three factors have significant effects on the total phenolic content to varying degrees, and there are also interaction effects. The substances that clear hydroxyl radicals are only affected by the ethanol concentration. This is a type of substance greatly influenced by polarity, while the substances that inhibit DPPH radicals are completely different, only affected by the liquid–solid ratio and extraction time. Moreover, there is a significant interaction effect between the ethanol volume fraction and the extraction time. These reasons also led to it being difficult for the final optimization results to take into account all three dependent variables. The subsequent purification process also confirmed this situation, as the two different types of active substances could not be obtained simultaneously.

Through software maximization analysis, the optimal scheme given is as follows: A certain mass of *Sargassum fusiforme* powder was weighed, and 29 times its volume of ethanol–water solution (28%) was added, that is, the liquid–solid ratio was 29 mL/g. The extraction system was maintained at 80 °C for 3.2 h before the extraction was completed. Under these conditions, an expanded validation experiment was conducted. The actual total phenolic content obtained was 5.06 ± 0.09 mg GAE/g. The relative scavenging rate of hydroxyl radicals by the crude SFP extract (50 mg/L) was 36.65 ± 0.92%, and the relative scavenging rate of DPPH radicals by the crude SFP extract (10 mg/L) was 62.71 ± 0.63%, which showed no significant difference from the predicted levels.

### 3.2. Process Optimization of SFP Enrichment with Macroporous Resin

**Figure 2:**
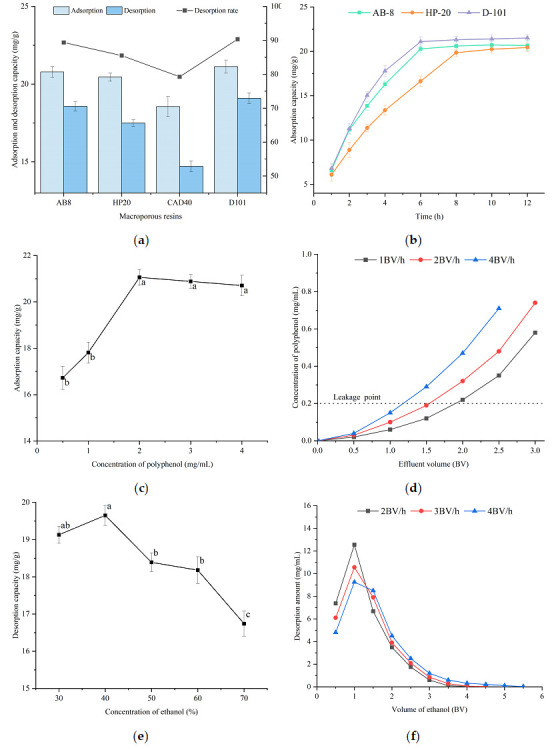
Process of SFP enrichment with macroporous resin. (**a**) Adsorption and desorption capacities of tested macroporous resins; (**b**) adsorption kinetics curves of tested macroporous resins; (**c**) effect of loading concentration of crude SFP extract on adsorption capacity; (**d**) leakage curve of different flow velocities; (**e**) effect of concentration of ethanol on desorption capacity; (**f**) dynamic desorption curve. The letters a–c indicated a significant difference (*p* < 0.05).

Four kinds of macroporous resins (AB-8, HP20, CAD40, D101) were screened and used in the optimization of the SFP enrichment process. Table 4 shows the models and parameters of four types of macroporous resins. They are all polymerized from styrene. Although they are non-polar materials, they have a separation advantage for structures such as phenols and flavonoids, which takes advantage of the non-covalent interaction of π-π packing formed by the aromatic rings of styrene and polyphenols. On this basis, the addition of substrate during the synthesis process can increase or decrease the polarity exhibited by the resin through different functional groups to adapt to different reaction environments. If a certain number of ester groups is involved in the synthesis of D101 resin, the hydrogen bond between the ester groups and the hydroxyl groups of polyphenols can enhance the adaptability of non-polar resins to the purification of polyphenols. Their static adsorption and desorption results are shown in Figure 2a. Obviously, CAD40 has a relatively low adsorption capacity and a desorption rate of less than 80%, while the saturated polyphenol capacity and desorption rate values of the other three macroporous resins are very close. AB-8, HP20, and D101 are all suitable for polyphenol purification. Further comparison and detailed analysis of the parameters show that CAD40 has the smallest pore size, which hinders the entry of large molecular polyphenols, allowing only small molecular phenols to be adsorbed. Coupled with its smallest specific surface area, its adsorption and desorption performances are both poor. HP20 has the largest specific surface area among the four resins, but it does not significantly increase the capacity. This might be related to the moderately polar resin material, which has poor selectivity and adsorbs impurities in the mixture, saturating the binding sites with impurities. Thus, a large capacity in parameters does not necessarily mean a higher polyphenol adsorption value. AB-8 and D101 are the two most suitable resins. Possibly due to D101 having a larger pore volume, its polyphenol adsorption capacity is slightly higher than that of AB-8, while the desorption rates of the two resins are almost the same. Based on this data comparison, it can be inferred that non-polar or weakly polar resins with a pore size greater than 10 nm are beneficial for the purification of polyphenols.

Since the results of the static adsorption and desorption experiments of the three types of macroporous resins, AB-8, HP20, and D101, were similar, the adsorption kinetics curves of the three were plotted for further detailed comparison (Figure 2b). Generally, the material transport mechanism of macroporous resins is considered to have multiple stages [25]: the diffusion of the target substance on the resin surface, the diffusion of the target substance from the surface to the interior of the macropores, and the capture of the target substance by the binding sites within the pores. The equilibrium time of HP20 is approximately 2 h slower than that of the other two resins. This may be caused by the smaller pore size restricting the diffusion of substances. AB-8 and D101 have larger pore sizes, and their equilibrium speeds are also relatively faster. Perhaps AB-8 has some polar advantages. At the beginning of adsorption for about 1 h, the velocities of the two resins are similar. However, as the diffusion process of substances progresses continuously, D101 with larger pore diameters and pore volumes appears to be more resilient, resulting in differences. Admittedly, the difference between AB-8 and D101 is very small; they both reached their maximum adsorption capacity at 6 h. In the study of the purification kinetics of related polyphenols, both satisfy the fit of the quasi-second-order kinetic model [26], which indicates that their mass transfer mechanisms are also similar. Considering that large-capacity resins are of greater significance for large-scale production, D101 macroporous resin was selected to conduct parameter optimization experiments.

Figure 2c shows the adsorption effect of the same polyphenol mass (40 mg) under different loading concentration conditions. A high concentration will accelerate the entry of polyphenols into macropores and speed up the adsorption process, reaching the peak at 2 mg/mL. When the concentration is too low or too high, impurities with similar properties in the crude SFP extract may compete for adsorption sites, resulting in a decrease in the adsorption capacity of polyphenols. Even if 40 mg of polyphenols, much higher than the saturation amount, is uniformly used in the experiment, this competitive effect is more obvious at low concentrations. It can be determined that loading the sample with the crude SFP extract at a concentration of 2 mg/mL has the best adsorption effect.

The adsorption capacity of macroporous resin for polyphenols varies at different flow rates. The flow rate represents the time cost. The significance of the leakage curve is to stop the sample loading in time when the concentration of the effluent is relatively high to prevent the waste of the sample solution. Usually, this value is defined as 10% of the concentration of the sample solution [27]. The corresponding concentration in this study was 0.2 mg/mL. As shown in Figure 2d, when the flow rate increases, the leakage point appears earlier. At a higher flow rate, the solid–liquid mass transfer time shortens, and the polyphenols are not adsorbed in time and flow out of the chromatographic column quickly. However, too low flow rates will increase the loss of time cost and affect production efficiency. The time required to flow out of the dead volume at flow rates of 1 BV/h and 2 BV/h is 1 h and 0.5 h, respectively, and the time required to reach the leakage point subsequently is approximately 1.8 h and 0.75 h, respectively. The difference in time consumption is very large. Although 4 BV/h is very fast, the corresponding sample loading volume is slightly higher than 1 BV and the volume is relatively small. Finally, a flow rate of 2 BV/h and a sample loading volume of 1.5 BV were selected for the subsequent experiments.

The determination of the volume fraction of ethanol is shown in Figure 2e. A 40% ethanol aqueous solution has a high desorption capacity, which can break the interaction between macroporous resin and polyphenols, allowing polyphenols to be released from the resin adsorption and enter the eluent. As the proportion of ethanol in water increases, the polarity of the mixture continuously decreases. Generally, polyphenols are considered to have high polarity. According to the principle of like dissolves like, the use of a higher polarity ethanol solution in the extraction process optimization conforms to this principle. This also leads to the phenomenon that the desorption effect of polyphenols in low-polarity solutions continuously decreases. Therefore, a 40% ethanol aqueous solution was subsequently selected for elution.

The influence of different flow rates on the dynamic desorption efficiency is shown in Figure 2f. The three flow rates (2 BV/h, 3 BV/h, and 4 BV/h) all exhibit similar trends, with approximately 2 BV being sufficient to desorb the majority of polyphenols. The difference lies in that the peak shape is taller at the lower flow rate, the desorption area is more concentrated, and the tailing is much shorter than that at the higher flow rate. During the desorption process at high flow rates, the contact time between the solvent and the resin is too short, resulting in insufficient desorption time, which is manifested as the need for a larger volume of ethanol solution to achieve a similar effect as at the lower speed. The desorption volumes for the three flow rates are approximately 3.3 BV, 4 BV, and 4.5 BV, respectively. Although the amounts of 40% ethanol solution used are different, the time spent on desorption is similar. The ethanol volume fraction in the desorption step has a significant impact on the results, while the flow rate has a relatively minor effect. Considering the economic principle in production, desorption speeds of 2 BV/h and 4 BV of 40% ethanol solution are adopted for desorption.

### 3.3. Characterization of SFPs

Table 5 shows the composition and purity of the main phytochemical components of the SFP extract. Four common antioxidant components (total polyphenols, flavonoids, polysaccharides, and fucose) were all detected, except for phytosterols. The purity of all these components significantly increased after enrichment. The crude SFP extract contained many impurities, resulting in a relatively low purity of polyphenols (1.20%). These impurities also led to a polyphenol adsorption capacity of only about 20 mg when the macroporous resin was saturated, which explains why this value is much lower than the saturation values reported in similar purification literature [28]. Since liposoluble compounds, polysaccharides, and proteins are typical impurities in the ethanol extract of polyphenols [16], they compete with polyphenols for binding sites, causing a lower adsorption capacity. After enrichment with D101 macroporous resin, the purity of total polyphenols increased approximately eight times, and the purity of flavonoids rose from 0.59% to 2.04%. Clearly, D101 macroporous resin is effective in targeted selection and enrichment of polyphenols.

A high content of polysaccharides was detected in the SFP extract. Polysaccharides are a high-content component in *Sargassum fusiforme*, and fucose is an important component of polysaccharides. The content of polysaccharides in *Sargassum fusiforme* far exceeds that of polyphenols [6,29], but in the SFP extract of this study, polysaccharides and polyphenols belong to the same order of magnitude. One of the reasons for this is that ethanol can cause polysaccharide precipitation [3]. The extraction process inhibited the extraction of polysaccharides through an appropriate ethanol concentration. Another reason is that the macroporous resin used in the purification process has a strong selectivity for polyphenols, which results in the polysaccharides not being enriched in large quantities. When the purity of polyphenols increases by approximately eight times, the purity of polysaccharides and fucose only improves slightly. The components in Table 4 also trigger some associations. Due to the interaction between flavonoids, phenolic acids, and polysaccharides present in the algae, the polarity of phenolic substances is increased [30]. The presence of these components may also be one of the reasons why the proportion of water in the ethanol solution used for polyphenol extraction is relatively large.

Table 6 presents the results of qualitative analysis of the enriched SFPs using HPLC-QQQ-ESI-MS/MS technology (Danaher Corporation, Shanghai, China). A total of 11 phlorotannins in three categories, 2 flavonoids, 5 carboxylic acids, and 2 fatty acids were identified, including phloroglucinol. Except for carboxylic acids and stearic acid, which have no special functions, the rest of the compounds are antioxidant functional components, such as dihydroxyisoflavone, methylcatechin, and arachidonic acid, which are all well-known antioxidant components.

In the SFP extract, except for fucols, three main subspecies of phlorotannins were identified. In some of the identification literature, fucols are less often found in Sargassum, while fuhalols are usually more common [7,31,32]. Similar to the subspecies distribution in the above-mentioned literature, six types of fuhalol-type phlorotannins were identified in the SFP extract in this study, namely, bifuhalol, trifuhalol, tetrafuhalol, hydroxypentafuhalol, hydroxyhexafuhalol, and dihydroxyheptafuhalol. The notable feature of fuhalols is the presence of ortho-trihydroxyl groups. Due to the more active electron transfer of ortho-trihydroxyl groups than that of m-trihydroxyl groups, fuhalols have a greater possibility of high activity and the possibility of generating high polymers [10]. Fucophlorethol-A and eckol have different chemical bonds but the same degree of polymerization. Their molecular weights only differ by 2, but their antioxidant capabilities vary significantly. In hydroxyl radical, superoxide radical, and enzyme experiments, the former is stronger than the latter, which leads to the still-unclear REDOX mechanism regarding the relationship between bond type, branching, and reactivity [33]. The compounds of *m*/*z* 621 have three possible structures, and the main differences between fucol, phlorethol, and fucophlorethol come from the chemical bonds. This is due to the limitations of mass spectrometry, which cannot determine the sequence and structure of phloroglucinol units [34]. The qualitative determination of this component requires further confirmation of its spatial distribution through NMR. Under the combined action of the various phlorotannins, flavonoids, and unsaturated fatty acids mentioned above, the SFP extract formed the final biochemical characterization. Without standard substances as references, the HPLC-QQQ-ESI-MS/MS method can only be used to conduct qualitative analysis and cannot obtain the specific content of each compound.

### 3.4. Antioxidant Activities of SFPs

**Figure 3:**
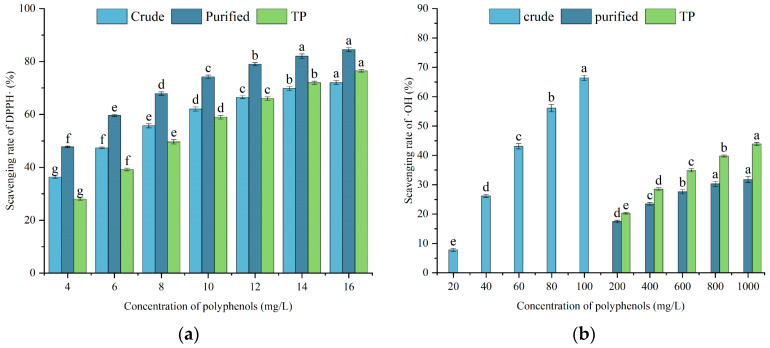

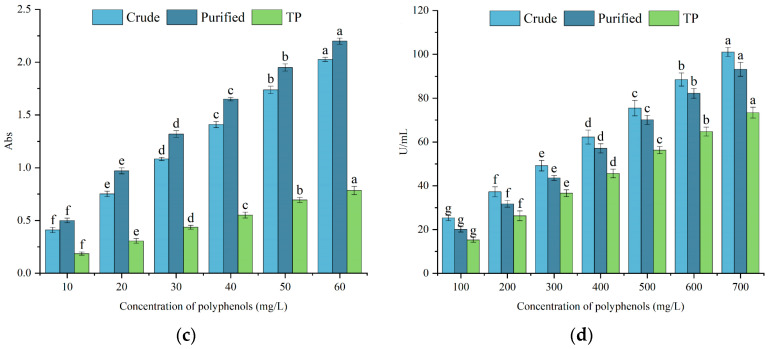
Determination of (**a**) DPPH radical scavenging activity, (**b**) hydroxyl radical scavenging activity, (**c**) reducing ability, and (**d**) total antioxidant capacity of SFPs. The letters a–g indicated a significant difference (*p* < 0.05).

Tea polyphenols are the general term for polyphenolic compounds in tea and have been proven to possess excellent biological activities such as antioxidation, anti-inflammation, and tumor prevention. Tea polyphenols are widely used as antioxidants in the food industry, animal husbandry, and disease treatment [35]. This study used purchased commercial tea polyphenols as a control to evaluate the antioxidant activity of SFPs. As shown in Figure 3a, the ranking of the strength of the free radical scavenging activity of the three components for DPPH is enriched SFPs > crude SFPs ≈ tea polyphenols. The scavenging power of enriched SFPs is the strongest, which may be accomplished by the more phenolic and other compounds in the same sample volume after enrichment. Crude SFPs and enriched SFPs have a similar trend, indicating that the components acting on DPPH free radicals are similar, but the purity is different.

Reactive oxygen species (ROS), such as hydroxyl radicals, superoxide anion radicals, hydrogen peroxide, etc., are conventional by-products in the respiratory chain reaction of human cells. Excessive ROS can damage cells and cause damage to cellular proteins and DNA as well as a series of diseases [36]. Therefore, evaluating the hydroxyl radical scavenging ability has practical significance. As shown in Figure 3b, the crude SFPs exhibit a hydroxyl radical scavenging ability far superior to that of the enriched SFPs and tea polyphenols. This may be related to a certain polar-sensitive component, which is likely to be removed during the enrichment of SFPs in macroporous resins, possibly terpenes or glycosides with significant structural differences from the polyphenols.

The determination principles of the Prussian blue method and the o-phenanthroline method are both based on the reduction of Fe^3+^ to Fe^2+^ by antioxidants. However, this study presented different effects, which also shows the significance of the need for multiple system evaluations of the total antioxidant capacity [37]. In Figure 3c, the order of reducing power strength among the three groups is enriched SFPs > crude SFPs > tea polyphenols. The overall trend is similar to the ability to eliminate DPPH free radicals, and the compounds involved in these two reactions should also have certain similarities. As shown in Figure 3d, the ranking of the strength of the total antioxidant capacity determined by the o-phenoline method is crude SFPs > enriched SFPs > tea polyphenols. This trend is similar to the hydroxyl radical scavenging ability. The reason for this phenomenon may still be related to the unknown polar-sensitive component. The enriched SFPs lose this component, resulting in some antioxidant indicators being weaker than those of the crude SFPs.

*Sargassum fusiforme* has the following advantages when used as an alternative source of tea polyphenols: (1) Brown algae polyphenols are natural products from the ocean, which meet consumers’ pursuit of “natural and healthy”. (2) *Sargassum fusiforme* grows rapidly, has a large biomass, and does not occupy arable land or freshwater resources, and its sustainable cultivation and harvesting also conform to the concept of environmental protection. The cultivation of tea trees requires the occupation of land resources and involves agricultural activities such as fertilization and pest control. (3) Compared with polyphenols from terrestrial plants, brown algae polyphenols have some unique chemical structures, which may imply that they possess distinctive mechanisms of action and biological activities. (4) Brown algae polyphenols have a larger molecular weight and more phenolic hydroxyl groups, which can provide more hydrogen atoms to neutralize free radicals. Meanwhile, the thermal sensitivity of SFPs should be given due attention. How to obtain a high yield and high activity of SFPs at lower temperatures, as well as maintain their stability during storage and application, are all issues that need to be deeply explored in further research.

## 4. Conclusions

In conclusion, this study established an extraction and enrichment process for SFPs with antioxidant activity that is suitable for large-scale production. The SFP extract mainly consists of polyphenols, flavonoids, and polysaccharides, and the phlorotannins it contains are mainly fuhalol-type. The enriched SFPs exhibited superior antioxidant activity compared to tea polyphenols in three antioxidant evaluation systems (DPPH free radical scavenging ability, reducing power, and total antioxidant capacity). The research results provide a theoretical basis and experimental evidence for the development of a new type of food preservative based on SFPs.

## Figures and Tables

**Table 1:** Factors, levels, and other conditions of the single-factor experiments.

Factors	Levels	Other Conditions
Ethanol volume fraction (%)	20, 30, 40, 50, 60	Liquid–solid ratio 20 mL/g, temperature 70 °C, time 4 h
Extraction temperature (°C)	50, 60, 70, 80, 90	Volume fraction of ethanol 30%, liquid–solid ratio 20 mL/g, time 4 h
Liquid–solid ratio (mL/g)	15, 20, 25, 30, 35	Volume fraction of ethanol 30%, temperature 80 °C, time 4 h
Extraction time (h)	1, 2, 3, 4, 5	Volume fraction of ethanol 30%, liquid–solid ratio 25 mL/g, temperature 80 °C

**Table 2:** Parameters and results of response surface models.

No.	*X*_1_	*X*_2_	*X*_3_	*Y*_1_	*Y*_2_	*Y*_3_
	Ethanol Volume Fraction (%)	Liquid–Solid Ratio (mL/g)	Extraction Time (h)	Total Phenolic Content (mg/g)	Hydroxyl Radical Scavenging Rate (%)	DPPH Radical Scavenging Rate (%)
1	−1 (20)	−1 (20)	0 (3)	4.27	39.09	60.00
2	+1 (40)	−1 (20)	0 (3)	4.75	30.23	60.65
3	−1 (20)	+1 (30)	0 (3)	4.81	38.78	60.02
4	+1 (40)	+1 (30)	0 (3)	4.90	31.95	62.86
5	−1 (20)	0 (25)	−1 (2)	4.46	33.76	57.48
6	+1 (40)	0 (25)	−1 (2)	4.69	29.03	59.48
7	−1 (20)	0 (25)	+1 (4)	4.82	35.00	61.78
8	+1 (40)	0 (25)	+1 (4)	4.77	29.89	59.63
9	0 (30)	−1 (20)	−1 (2)	4.28	35.41	59.44
10	0 (30)	+1 (30)	−1 (2)	4.96	34.42	59.62
11	0 (30)	−1 (20)	+1 (4)	4.80	34.41	61.14
12	0 (30)	+1 (30)	+1 (4)	4.99	34.09	63.11
13	0 (30)	0 (25)	0 (3)	5.08	35.96	63.06
14	0 (30)	0 (25)	0 (3)	5.10	37.53	62.40
15	0 (30)	0 (25)	0 (3)	5.03	37.69	63.55
16	0 (30)	0 (25)	0 (3)	4.96	36.58	62.34
17	0 (30)	0 (25)	0 (3)	5.09	35.94	63.02

**Table 3:** Analysis of variance for extraction models.

Source	*Y*_1_ (Total Phenolic Content)			*Y*_2_ (Hydroxyl Radical Scavenging Rate)			*Y*_3_ (DPPH Radical Scavenging Rate)		
	F Value	*p* Value	Sig.	F Value	*p* Value	Sig.	F Value	*p* Value	Sig.
Model	19.17	0.0004	**	11.08	0.0022	**	13.22	0.0013	**
*X*_1_	11.70	0.0111	*	60.21	0.0001	**	3.57	0.1008	
*X*_2_	50.15	0.0002	**	0.0007	0.9789		6.04	0.0437	*
*X*_3_	19.87	0.0029	**	0.056	0.8194		29.44	0.0010	**
*X*_1_ *X*_2_	6.28	0.0407	*	0.76	0.4118		3.09	0.1223	
*X*_1_ *X*_3_	3.26	0.1137		0.027	0.8749		10.89	0.0131	*
*X*_2_ *X*_3_	9.94	0.0161	*	0.080	0.7849		2.00	0.1999	
*X*_1_^2^	33.35	0.0007	**	15.01	0.0061	**	27.76	0.0012	**
*X*_2_^2^	15.58	0.0055	**	0.68	0.4356		1.55	0.2527	
*X*_3_^2^	15.22	0.0059	**	21.42	0.0024	**	29.59	0.0010	**
Lack of Fit	0.2061			0.1467			0.2219		
*R* ^2^	0.9610			0.9344			0.9444		
Adj *R*^2^	0.9109			0.8500			0.8730		
C.V.%	1.62			3.35			1.03		

* *p* < 0.05; ** *p* < 0.01.

**Table 4:** The models and parameters of four types of macroporous resins.

Macroporous Resin Model	Pore Volume (ml/g)	Specific Surface Area (m^2^/g)	Aperture (nm)	Polarity
AB-8	0.73−0.77	480−520	13−14	Weak polarity
HP20	1.1−1.3	550−600	9−10	Medium polarity
CAD40	0.73−0.77	450−500	7−8	Weak polarity
D101	1.18−1.24	480−520	25−28	Non-polarity

**Table 5:** Components and purities of the SFPs before and after enrichment.

Procedure	Polyphenols (%)	Flavonoids (%)	Polysaccharides (%)	Fucose (%)	Phytosterols (%)
Before enrichment	1.20 ± 0.08 ^b^	0.59 ± 0.04 ^b^	4.65 ± 0.11 ^b^	1.06 ± 0.07 ^b^	Not detected
After enrichment	10.78 ± 0.25 ^a^	2.04 ± 0.35 ^a^	6.43 ± 0.20 ^a^	1.86 ± 0.13 ^a^	Not detected

The letters a and b indicated a significant difference (*p* < 0.05).

**Table 6:** Analysis of compounds in the enriched SFPs by LC-MS/MS.

Classification	RT (min)	[M−H]^−^ (*m*/*z*)	MS/MS Ions (*m*/*z*)	Tentative Assignment
Phenols	1.15	125	97	Phloroglucinol
Fuhalols	3.04	265	111, 123, 125, 139, 141, 247	Bifuhalol
	1.06	389	125, 139, 245, 265	Trifuhalol
	1.89	513	246, 265, 373, 389	Tetrafuhalol
	1.46	653	245, 263, 387, 389, 513	Hydroxypentafuhalol
	2.27	777	245, 387, 389, 513	Hydroxyhexafuhalol
	8.38	917	527, 653, 785	Dihydroxyheptafuhalol
Fucol/phlorethol/fucophlorethol	1.35	621	247, 263, 355, 373, 495	/
Fucophlorethols	1.08	373	233, 229, 125	Fucophlorethol-A
Eckols/carmalols	3.04	263	111, 219, 245	Carmalol
	3.09	387	123, 245, 262	Phlorethoxycarmalol
	2.70	371	121, 140, 229, 246	Eckol
Flavonoids	1.30	253	225, 197, 143	4’,7-Dihydroxyisoflavone
	4.63	303	271, 163	3’-O-Methylcatechin
Carboxylic acids	1.33	135	117, 91	Threonic acid
	1.30	221	193, 149, 121	Diethyl phthalate
	3.77	339	321, 295, 211	Behenic acid
	0.89	157	139, 113, 97	3-Oxooctanoic acid
	2.70	251	233, 207, 165	Mono-(3-carboxypropyl) phthalate
Fatty acids	2.27	283	265, 240, 237	Stearic acid
	5.88	303	259, 285, 259	Arachidonic acid

## Data Availability

The original contributions presented in the study are included in the article; further inquiries can be directed to the corresponding authors.
